# Payment Reform: Unprecedented and Evolving Impact on Gynecologic Oncology

**DOI:** 10.3389/fonc.2016.00084

**Published:** 2016-04-15

**Authors:** Sachin M. Apte, Kavita Patel

**Affiliations:** ^1^Department of Gynecologic Oncology, Moffitt Cancer Center, Tampa, FL, USA; ^2^The Brookings Institution, Washington, DC, USA

**Keywords:** physician payment reform, gynecologic oncology, MACRA, MIPS, alternative payment models

## Abstract

With the signing of the Medicare Access and CHIP Reauthorization Act in April 2015, the Centers for Medicare and Medicaid Services (CMS) is now positioned to drive the development and implementation of sweeping changes to how physicians and hospitals are paid for the provision of oncology-related services. These changes will have a long-lasting impact on the sub-specialty of gynecologic oncology, regardless of practice structure, physician employment and compensation model, or local insurance market. Recently, commercial payers have piloted various models of payment reform via oncology-specific clinical pathways, oncology medical homes, episode payment arrangements, and accountable care organizations. Despite the positive results of some pilot programs, adoption remains limited. The goals are to eliminate unnecessary variation in cancer treatment, provide coordinated patient-centered care, while controlling costs. Yet, meaningful payment reform in oncology remains elusive. As the largest payer for oncology services in the United States, CMS has the leverage to make cancer services more value based. Thus far, the focus has been around pricing of physician-administered drugs with recent work in the area of the Oncology Medical Home. Gynecologic oncology is a unique sub-specialty that blends surgical and medical oncology, with treatment that often involves radiation therapy. This forward-thinking, multidisciplinary model works to keep the patient at the center of the care continuum and emphasizes care coordination. Because of the breadth and depth of gynecologic oncology, this sub-specialty has both the potential to be disrupted by payment reform as well as potentially benefit from the aspects of reform that can align incentives appropriately to improve coordination. Although the precise future payment models are unknown at this time, focused engagement of gynecologic oncologists and the full care team is imperative to assure that the practice remains patient centered, embodies the highest quality in research and education, yet transforms into a sustainable and agile sub-specialty to pro-actively and effectively manage the immense and relentless financial pressures and regulatory expectations that will be faced over the next decade.

## Introduction

On April 16, 2015, President Barack Obama signed the Medicare Access and CHIP (Children’s Health Insurance Program) Reauthorization Act (MACRA). This new legislation repealed the ineffective and maligned sustainable growth rate (SGR) mechanism of updating fees to the physician fee schedule (1, 2). As the policies within MACRA are implemented, they will significantly impact reimbursement and care delivery for oncology services. Many payment reform models piloted thus far have primarily focused on primary care and hospital-based episodes of care with recent pilots in medical oncology. These models vary in the extent to which physician services are aggregated across providers and the degree to which payments are distributed across different settings. Examples include the use of a modified pay-for-performance or a fee for the use of disease specific oncology pathways, bundled payments, oncology patient-centered medical homes (PCMHs), episode payment for services, and accountable care organizations (ACOs). The sub-specialty of gynecologic oncology is unique; physicians frequently function as both the surgeon as well as the medical oncologist. They often coordinate other modalities of therapy, such as radiation, and frequently remain the primary coordinator of their patients’ cancer care team throughout the trajectory of their disease. This includes those patients who transition to hospice. The nature of the training of gynecologic oncologists yields important efficiencies in terms of care coordination and potential reduction in unnecessary treatments or duplicative testing. Due to the breadth and depth of the subspecialty, physician payment reform will significantly impact the practice of gynecologic oncology. In this paper, we will first review some historic and current methods to achieve payment reform. Then we will review the preliminary details and implications of MACRA and discuss the possible profound and long-lasting effects on gynecologic oncology.

### Historic and Current Components of Payment Reform

Currently, fee-for-service (FFS) is the most common reimbursement methodology in oncology despite efforts to implement alternative approaches. This form of payment can inadvertently incent high-volume, high-cost procedural services. FFS often undervalues or fails to reimburse evidence-based, cost efficient, effective services such as patient education, prevention, care coordination, or end-of-life discussions. As an unintended consequence, these perverse incentives can lead to fragmentation, inefficiency, and waste. Payment reform in the FFS system previously has consisted of pay-for-performance (P4P) programs that have usually been a variation of FFS payments with a bonus element added for achieving certain quality milestones. Historic quality contracts have generally been a P4P model with limited success.

The care of women with gynecologic malignancies in the United States has greatly improved over the last several decades. A recent study demonstrated an improvement in relative survival for all stages of ovarian cancer from 1975 to 2011 (3). Possible reasons for this beneficial trend include the recognition of the importance of surgical staging and cytoreductive procedures; platinum and taxane-based therapy; intra-peritoneal chemotherapy; and the development of other effective chemotherapeutic and biologic agents. A population-level analysis from 1983 to 2009 showed an improvement in relative survival for women with stages I–III cervical cancer (4). A recent study demonstrated an improvement in overall survival in patients with recurrent, persistent, or metastatic cervical cancer with the addition of bevacizumab to combination chemotherapy (5). There was no significant deterioration in health-related quality of life in patients receiving anti-angiogenic therapy (6). Such advancements in cancer survival and maintenance of quality of life are predicated on scientific research. Efforts in both academia and industry have yielded progress in the understanding of the mechanisms of cancer prevention and treatment, paving the way to novel therapies that translate into improved outcomes. Due in part to the success in cancer therapy, increasing demand, and the demographics of an aging population, cancer care will remain a major driver of escalating healthcare spending in the United States. In the United States, approximately 1.6 million people are diagnosed with cancer annually. A 2011 study projected total cancer spending to be approximately $157 billion in 2020 – a 27% increase from 2010 (7). “The distribution of total cancer care costs is 32% for chemotherapy drugs, administration, and radiation; 33% for inpatient and physician surgical claims; and 12% for other physician services. The remaining 22% is composed of evaluation and management, hospice, laboratory tests, imaging services, and inpatient stays without surgery” (8). Due to the broad range of services provided by many gynecologic oncologists, the sub-specialty contributes to numerous different categories contributing to the total cost of cancer care. Therefore, the impact of payment reform on gynecologic oncology could be significant.

#### Sustainable Growth Rate

The SGR was a method previously used by the Centers for Medicare and Medicaid Services (CMS) that was designed to control spending on physician services. Enacted by the Balanced Budget Act of 1997, the SGR was designed to ensure that the annual increase in the expense per Medicare beneficiary did not exceed the growth in the Gross Domestic Product. The SGR formula was responsible for determining the annual increases or decreases to the Medicare physician fee schedule. Under the SGR mechanism, if the growth in the volume of services exceeded the target growth rate, the yearly update to fees was to be reduced with a “conversion factor” to bring spending in line with the target. The short-term fixes imposed administrative burdens on CMS and clinicians and they created uncertainty for health care professionals and beneficiaries about uninterrupted access to care (9). The resulting instability and uncertainty led to 17 overrides of scheduled fee cuts. The repeal of the SGR now means that the temporary measures to override the growth rate formula will no longer dominate Medicare policy discussions, as they have for the last decade. The replacement of the SGR should also accelerate the movement away from unconstrained FFS payments and toward continued payment reforms.

#### Physician Quality Reporting System

The Physician Quality Reporting System (PQRS) is a voluntary quality reporting program established by CMS in 2007, which follows a P4P model – namely that physicians are paid a fraction of their FFS payments initially as a positive bonus on their overall reimbursable claims. The program was designed to encourage both individual providers as well as group practices to report quality of care data to Medicare (10). PQRS provides the opportunity to assess the quality of care provided by a physician or practice and quantify their performance on a particular metric. Beginning in 2015, if an eligible professional or group practice did not satisfactorily report PQRS measures in 2013, they would receive a 1.5% payment penalty on their 2015 Medicare reimbursements. Providers and practices who report in a compliant manner for the 2015 program year will not receive the 2017 PQRS negative payment adjustment. The Society of Gynecologic Oncology (SGO) has published PQRS measures relevant to gynecologic oncology (11).

#### Electronic Health Records Incentive Programs (“Meaningful Use”)

The electronic health record (EHR) incentive program was established as part of the American Recovery and Reinvestment Act of 2009 (ARRA). ARRA amended the Social Security Act by creating incentive payments to providers and hospitals “to promote the adoption and meaningful use (MU) of interoperable health information technology (HIT) and qualified EHRs. These incentive payments are part of a broader effort under the HITECH (Health Information Technology for Economic and Clinical Health) Act to accelerate the adoption of HIT and utilization of qualified EHRs” (12). “MU” has three stages that began in 2011. The objective of Stage 1 (2011–2012) was to promote basic EHR adoption, data capture, and sharing. For Stage 2 (2014), the objectives were to advance clinical processes and emphasize care coordination and the exchange of patient information. Stage 3 is expected to be implemented in 2016 with a goal to show that the quality of health care has been improved.

#### Physician Value-Based Payment Modifier

The Affordable Care Act (ACA) directs the Secretary of Health and Human Services (HHS) to develop and implement a budget-neutral process to financially reward physicians who provide health care that is high in quality and low in cost (13). This system, the physician value-based payment modifier (PVBM), will adjust the fee schedule payments based on the quality and cost of care delivered to Medicare beneficiaries. “The PVBM reward formula is a system in which performance is assessed in two dimensions (quality and cost), with payments accruing to physicians who have above-average performance along both dimensions. Physicians who perform worse than average or choose not to be involved will be paid less, while those with average performance will experience no change” (14). The maximum bonus is ~2% of Medicare fees and the maximum penalty is ~1%, based on the 2013 program year (13). Thus, the model is similar to a P4P-type program with the majority of payments in a traditional FFS setting and a smaller bonus or penalty based on performance. When defining PVBM, “CMS will use the PQRS quality measures reported by individual physicians and by groups under that program’s reporting mechanism of which there are several options. Total per capita costs for Medicare beneficiaries will be used to calculate a cost composite score for the value-based payment modifier” (14).

## Medicare Access and CHIP Reauthorization Act of 2015 ERA

When Congress passed the MACRA, it gave HHS the authority to move ahead with alternative payment models (APMs). MACRA introduced comprehensive changes to how Medicare pays physicians and hospitals for among many areas, oncology-related services. MACRA makes three important changes to how Medicare pays healthcare providers who care for Medicare beneficiaries. First, the new law repeals the SGR formula as a mechanism for determining Medicare payments for physicians’ services and puts into place a predictable annual increase through 2019 before a complete transition to the new system described below. Second, MACRA establishes two payment options beginning in 2019, which create a new framework for rewarding providers for giving better care and not simply more care. One option is the merit-based incentive payment system (MIPS) that consolidates current programs and retains many elements of the current FFS structure with a new system for positive or negative adjustments to the fee schedule payments. Critics have argued that in many ways, it is largely a P4P-type model and many physicians who are either confused or intimidated by APMs will chose MIPS and potentially tolerate penalties and flat/negative payment adjustments in medicare. The second option, participation in an APM, is different from the current FFS system. Both choices move toward a valued-based system, with an overarching emphasis on quality, not volume, of healthcare services provided. Regardless of the pathway within MACRA, the new reimbursement system will likely require transformative changes to the structure of a medical practice. Both paths require practices to (1) report quality metrics, (2) demonstrate MU of EHRs and use resources responsibly, and/or (3) take on financial risk. Third, MACRA incentivizes practice transformation by combining the existing quality reporting programs into one new system. While many have hailed the repeal of the SGR mechanism, the passage of MACRA now raises new questions about where the United States health care system is headed in the post-SGR world of payment and delivery reform.

### Merit-Based Incentive Payment System

The MIPS is a new payment system that consolidates existing P4P programs and accounts for quality, resource use, EHR utilization, and clinical practice improvement. MIPS combines parts of the PQRS, the PVBM, and the MU program – and adds a new category of clinical practice improvement activities – into a single program that will assess physicians on these categories. The MIPS Composite Score will include components for quality (approx. 30% based on PQRS by 2021), MU (initially 25%, then reduced to 15%), resource use (30% based on PVBM by 2021), and clinical practice improvement (25%). Clinical practice improvement activities are those that contribute to advancing care coordination, safety, and care. Examples include expanding access, care coordination, safety, and participation in registries. Although details on MIPS will be the subject of policymaking for several years, it is important to understand that some of the assessments made at the effective date of 2019 will be based upon 2017 data. For the 2015 and 2016 performance years, the PQRS, PVBM, and MU programs will continue as separate payment adjustment programs. MACRA provides physicians and other health care professionals with stable fee updates for 5 years (an update of 0.5% for the last 6 months of 2015 and an increase of 0.5% per year for 2016 through 2019). For 2015 to 2018, the current payment system remains unchanged. Under MIPS, the payment rates in 2019 will be maintained through 2025 but with positive and negative adjustments based on the composite performance score of each eligible physician or other health professional on a 0- to 100-point scale. The scores will be publicly reported on the CMS Physician – Compare website. The composite score will be reported for all providers, compared to peers, and will be available to consumers. The adjustments, however, are designed to be budget neutral so that there would be no effect on overall payments beyond an additional $500 million that would be made available each year from 2019 to 2024 to reward excellent performance (15). The MIPS payment adjustments can be significant (±9% adjustments) with top performers earning +27%.

### Alternative Payment Models

The leadership at the Department of HHS aims “to have 30% of Medicare payments tied to quality or value through alternative payment models by the end of 2016 and 50% of payments by 2018” (16). Under the new legislation, clinicians who receive a substantial portion of their revenues from approved APMs will not be subject to MIPS. Instead, they will receive a 5% bonus each year from 2019 to 2024. To qualify, the APM must comprise 25% of provider revenue or patients between 2019 and 2020. By 2023, this increases to 75% of provider revenue or patients. In 2026, the payment rules for all clinicians change again, with payment rates under the APM increasing by 0.75% per year and rates for others increasing by 0.25% per year. MACRA incentivizes participation in APMs by establishing a system in which, beginning in 2019, qualifying healthcare providers may receive a lump sum for participation in a certified APM at a certain level. That incentive payment will be equal to 5% of the prior year’s estimated aggregated expenditures under the fee schedule. Beginning in 2026, when the lump sum payment goes away, the baseline fee schedule payments will still be higher for qualifying APM participants than for other providers in the MIPS system. APMs must involve a downside risk and quality measurement. While, currently, there are not many APMs for oncology, the legislation encourages development and recognition of models available to medical specialists, such as oncologists. How an APM will be recognized for purposes of the program is still evolving, but may include existing models, such as ACOs, PCMHs, and bundled payment models. MACRA also introduces a new pathway to qualify APMs, called physician-focused payment models (PFPMs). While CMS will determine which PFPMs qualify as an APM under MACRA, the law mandates that qualifying PFPMs require the reporting of quality measures, the use of certified EHRs, and that the physician has “more than nominal financial risk” (with the exception of a PCMH, for which the risk requirement is waived). Stakeholders can submit proposals. A newly established Technical Advisory Committee will assess PFPM proposals from stakeholders and make recommendations to the HHS Secretary about which models to adopt as a qualifying APM and the Secretary will consider and release a list of available APMs. The Secretary must release criteria for a qualifying APM by November 1, 2016.

### Approaches to the Design of Oncology-Focused APMs

Potential designs for APMs may be viewed along a continuum through greater bundling across either providers or payments. “APMs transition from volume- to case-based payments, reduce or limit the FFS component, and use performance measures to hold providers accountable. Providers gain flexibility by decoupling provider payments from the volume and intensity of specific services, but they also face greater accountability for lowering costs, and depending on the performance measures that affect payment, for better quality care and better results” (8). The ability of an APM to improve outcomes also depends on investments and support, such as the timely collection and analysis of validated data, systematic processes for data-driven learning, and deployment of user-friendly HIT systems. The success of more transformative APMs – including oncology PCMHs and oncology ACOs – will require greater investments in human resources, work flow changes, provider engagement, and other aspects of practice change, such as strategies to increase scale and deployment of sophisticated cost accounting tools. Successful implementation of a PCMH or an ACO will also impose a heavier administrative burden compared to clinical pathways or bundled payment models.

The specific type of APM implemented will also depend on the physician employment structure. The spectrum of an oncology provider’s employment model spans from single-specialty private practice, multi-specialty independent group practice, to hospital-based employment in a comprehensive cancer center or large regional multi-hospital system. In each scenario, there will be variation in the extent of physician alignment and the ability to bundle professional and technical charges. Although smaller practices may be more agile to respond to change, large-scale operations can reduce costs via efficiency, controls, standardization, and supply chain management. A large-scale health care system, offering a broad range of services across the care continuum, may also be more adept at retaining a patient throughout their oncology journey. In addition to medical, surgical, and radiation oncology-related services, this could include emergency room visits, home health, palliative care, and other medical specialty services across a large geographic area. Therefore, factors, such as physician integration, scope of services offered, and the scale of the health care enterprise, will significantly impact the decision of which APM is most appropriate in any local market.

A recent paper provides an outline of four different APMs – clinical pathways, oncology PCMHs, bundled payments, and oncology ACOs – to show a continuum of payment incentives that can influence the extent to which care delivery changes limit or reduce costs (8). These APMs were selected “because they can support incremental to comprehensive clinical transformations, thereby accounting for the breadth and size of oncology practices, populations served, and payer types.” These reforms, summarized in Table 1, can be viewed as building blocks along the spectrum of payment reforms.

**Table 1:** **Comparison of oncology payment models by delivery, physician employment, and payment structure, and quality measurement**.

Payment model		Clinical pathways	Oncology PCMH	Bundled payment	Oncology-specific ACO	Global payment
**Delivery structure**	Use of evidence-based pathways or guidelines	Yes	Yes	Yes	Yes	Yes
	Care coordination focus	No	Yes	Yes	Yes	Yes
	Requires major practice transformation	No	Yes	No (for the types of bundles currently in market)	Yes	Yes
**Probability of implementation based on physician employment structure**	Single-specialty group, private practice	Medium	Low	Low	Low	Low
	Multi-specialty group, private practice	Medium	Medium	Low	Low	Low
	Hospital employed, single general hospital	High	Medium	Medium	Low	Medium
	Hospital employed, comprehensive cancer center	High	High	Medium	Medium	Medium
	Hospital employed, multi-hospital system	High	High	High	High	High
**Payment structure**	Case-based payment component	Revenue neutral supplemental payment for pathways adherence	PMPM management fee	Episode-based prospective or retrospective payment for pre-determined defined bundle of service	Partial capitation	Total capitation
	Transition from P4P to value-driven care	P4P	P4P	Value driven	Value driven	Value driven
	Potential for global or capitated payment	No	No	Partially based on boundary of bundle (i.e., inpatient, imaging, ancillary service, etc.)	Yes	Yes
	Payment majority linked to quality and financial performance outcomes	No	No	Yes	Yes	Yes
**Quality measurement**	Incentives for continuous quality improvement activities	No	Yes	Yes	Yes	Yes
**Pilot programs**		Alabama Health Improvement Initiative, Oncology Clinical Pathways Pilot (17) and The WellPoint Cancer Care Quality Program (18)	New Mexico Cancer Center (19) and Wilshire Oncology Medical Group (20), Cancer & Hematology Centers of Western Michigan (21), Consultants in Medical Oncology and Hematology (22), and COME HOME, Moffitt Cancer Center and Aetna Oncology Medical Home Collaboration (23)	MD Anderson and UnitedHealthcare pilot in Head & Neck Cancer (24), Mobile Surgery International and BCBS of Florida (25), and Humana and 21st Century Oncology (26)	Florida Blue and Moffitt Cancer Center (27) and Baptist Health South Florida, Florida Blue and Advance Medical Specialities (28)	All-Payer Innovation Model in State of Maryland (29)

*Adapted from KP’s original work (8)*.

*Patient-centered medical home (PCMH), accountable care organization (ACO), pay for performance (P4P), per member per month (PMPM)*.

#### Clinical Pathways

Oncology-specific clinical pathways are standardized, evidence-based, dynamic, cost-effective protocols for the treatment of cancer patients. Although development, implementation, and assessment of compliance are challenges, pathways require comparatively limited structural changes to a practice or provider risk (30). This model is designed to encourage providers to adhere to disease-specific oncology pathways while reaching or exceeding quality benchmarks. An additional case management fee may be necessary to off-set the administrative burden of assessing pathway compliance and pathway maintenance. Early results show that pathway programs can decrease cost growth through diminished use of aggressive treatments that are not supported by clinical guidelines (31). Two papers demonstrate that pathways can reduce variation in chemotherapy use, while maintaining overall survival rates (32, 33). Reducing unnecessary clinical variation and providing more predictable costs is another goal of these reforms. Pathways alone, however, may not have a significant impact on care coordination or other aspects of personalized care. For some oncologists, pathways may represent an initial foray into practice standardization and assessment of compliance with evidence-based practice. Depending on the extent of physician integration and practice structure, proactive change management and a realistic assessment of local culture will be imperative to set the pace for pathway implementation and ultimately impact the likelihood of sustainable compliance.

#### Oncology Patient-Centered Medical Home

“The oncology PCMH is a practice-level approach that promotes care coordination and improvement through payments that are more extensively aligned with practice features expected to improve patient outcomes and patient-level performance measures” (8). Providers can use a per beneficiary per month fee in an oncology PCMH to support services that have traditionally not been reimbursed (i.e., access through expanded office hours, team-based care models, and advanced HIT) to encourage better patient education and care coordination and management (34). A successful implementation of an oncology PCMH would likely require an engaged and well-integrated group of oncology teams spanning a variety of sub-specialties beyond oncology. Improved care coordination combined with robust support for cost-effective services in the oncology PCMH model potentially reduces hospitalizations and emergency department (ED) visits, prevents overutilization of unnecessary high-cost drugs and services, and improves symptom management beyond the hospital setting (34, 35). Preliminary results from one oncology PCMH showed reductions in ED visits (68%), hospital admissions per patient treated with chemotherapy (51%), length of stay for admitted patients (21%), overall outpatient visits (22%), and outpatient visits in the chemotherapy population (12%) (34, 36). Although the increased administrative burden may erode margins, successful oncology PCMH models have reported significant net cost reductions via reduced ED visits and hospitalizations. One oncology PCMH reported aggregated savings of approximately $1 million per physician per year (37). Another program also saw substantial cost reductions from lower utilization of hospital admissions (34%), hospital days (44%), and ED visits (48%) (35). However, these savings have not been reproduced in all cases. The physician compensation model, practice structure, healthcare market, and local payer strategy may influence the level of adoption of an oncology PCMH. More evidence is required relevant to oncology PCMHs to determine specific factors of the payment and delivery reforms that may improve the likelihood of success.

#### Bundled Payment

A more comprehensive bundled payment methodology is possible, either within or outside an oncology PCMH. Providers are generally compensated with a one-time payment for a specific set of cancer services over a pre-determined treatment period or episode of care (32). “To the extent a broader range of services are bundled, providers can gain even more flexibility to redirect resources to cost-effective patient-centered activities that FFS does not reimburse” (8). The provider subsequently incurs greater accountability and more pressure to reduce the cost of care (38, 39). Recent results from one bundled payment pilot show a 34% reduction in total cost of care (40).

The extent of coverage of a bundled payment in oncology can vary based on how the bundle is designed. A bundle could be based on a timeframe; or within a pre-specified boundary on a pathway; or include technical and/or professional charges. Many other factors will need to be pre-determined. For example, how are costs allocated when care is delivered outside the scope of the agreement or in non-contracted facilities and labs? Most early pilots are limited bundles that included the administration of chemotherapy and supportive-care drugs (32, 39, 41, 42). More comprehensive bundles may include the drug acquisition costs, imaging and lab services, surgery, or radiation therapy. Bundled payments must be linked to performance benchmarks. To date, there are few prospective total cost bundles; there is a ground-breaking pilot program in head and neck cancer that bundles the total cost of care for 1 year in a prospective payment (24). The increased probability for cost variation per patient that accompanies more comprehensive bundles would imply that providers face more uncertainty about their net revenues. This likely explains why more comprehensive bundles have not been widely adopted to date. Another barrier to adoption is the challenge of cost accounting in a complex health care system. Factors such as physician integration and the ability of a hospital to fully cost expenses (i.e., labor, overhead, pharmacy, supplies, etc.) may vary widely. Therefore, there will likely be significant variation in the level readiness of oncologists and hospitals to move forward with bundled payments. At least initially, there may be significant variation in the scope and cost of a bundle based on local and regional factors. In the future, as more meaningful quality metrics become publically available, bundle payments may drive consumerism and competition.

#### Oncology-Specific Accountable Care Organization

The oncology ACO model partially links reimbursement to overall costs and quality of care for patients with cancer. In comparison, a “shared savings” oncology ACO would provide an incentive beyond the usual FFS payments, based on whether total spending for the relevant patients is below a benchmark and whether quality measures meet the pre-determined threshold. In an ACO environment, providers are accountable for the cost, quality, and overall care for a population in exchange for the opportunity to share savings with the payer. Therefore, the ACO construct encourages proactive management to deliver efficient, coordinated, and cost-effective cancer care. The increased accountability places critical importance on the administrative component of the ACO to manage and coordinate care thoughtfully. For this reason, some practices will require a significant transformation of practice and additional resources to participate as an oncology ACO.

Given the increasingly personalized, costly, and highly variable nature of oncology care, traditional ACOs have taken a measured approach toward oncology-specific reforms. Despite these challenges, there are several pilots of oncology-specific ACO arrangements and oncology-focused arrangements within population wide ACOs. These models link payment to performance metrics (Table 1). “Such oncology ACOs may also be partially or fully capitated, with some or all of the FFS payments related to oncology shifted into a fixed, risk-adjusted payment per patient that is contingent on meeting performance benchmarks. The extent to which an oncology ACO model resembles a global payment depends on the size and scope of the shift from FFS to a fully bundled capitation payment and whether other specialties are included” (8). The oncology ACOs remain in the early phase of development, but they are on a path of increasing clinical and financial risk (27, 43). To date, there is one global payment pilot (29) in which the case-based payment is totally capitated. In this innovative model, Maryland and CMS will evaluate an all-payer system for hospital payment. Payment will be based on Medicare per capita total hospital cost growth. In exchange, Maryland will be accountable to generate a pre-determined cost savings while achieving quality targets in the domains of readmission, hospital acquired conditions, and population health. Oncology services would be included in this pilot.

## Medicare Oncology Care Model

Centers for Medicare and Medicaid Services is developing novel payment and care models with the goal of improving the effectiveness and efficiency of specialty care. In February 2015, CMS’s Center for Medicare and Medicaid Innovation (CMMI) introduced a new payment and practice reform model, the oncology care model (OCM). The OCM is an innovative model for physician practices administering chemotherapy. Under the OCM, practices will enter into payment arrangements that include financial and performance accountability for episodes of care surrounding chemotherapy administration to cancer patients.

The goal of the OCM is to utilize appropriately aligned financial incentives to improve care coordination, appropriateness of care, and access to care for beneficiaries undergoing chemotherapy. The OCM encourages participating practices to improve care and lower costs through an episode-based payment model that financially incentivizes high-quality, coordinated care. Practitioners in an OCM are expected to rely on the most current medical evidence and shared decision-making. The OCM encourages commercial payers to participate in alignment with Medicare to create broader incentives for care transformation at the physician practice level. Other payers would also benefit from savings, better outcomes for their beneficiaries, and information gathered about care quality.

## Impact on Gynecologic Oncology

Much is still unknown in terms of the details of the MIPS and APMs and the subsequent impact on gynecologic oncology. The majority of current efforts to address new payment models focus heavily on medical oncology. In medical oncology, examples of pilot programs include CMMI’s Oncology Care Model (44) and Regional Cancer Care Associates and Horizon BCBS (45). The latter pilot focuses on bundled payments for breast cancer patients treated with chemotherapy. There are a limited number of APMs for surgical oncologic procedures. Mobile Surgery International and BCBS of Florida have designed a bundled payment for radical prostatectomy for early-stage patients (25). In radiation oncology, 21^st^ Century Oncology and Humana have developed a bundled payment for radiation therapy for 13 prevalent diagnoses, including breast, lung, and prostate cancers (26). On January 1, 2015, MD Anderson partnered with UnitedHealthcare in a pilot bundle in the treatment of head and neck cancers (24). This pilot program prospectively covers the total cost of care for head and neck cancer for 1 year. The sub-specialty of gynecologic oncology is unique, in that many gynecologic oncologists practice both surgical and medical oncology. In addition, the diseases managed by gynecologic oncologists frequently use radiation therapy, either in the primary, adjuvant, or palliative settings. For these reasons, the sub-specialty of gynecologic oncology is especially exposed to the unprecedented and evolving changes in physician payment reform. Due to the breadth of practice for many gynecologic oncologists, coupled with the heterogeneity of employment structure, APMs must be designed thoughtfully while embedding flexibility and equity. Due to the multitude of variables that require careful consideration and the variability in stakeholders, the optimal APMs will likely be designed locally.

In November 2015, the SGO submitted a request for information regarding the implementation of MIPS and the eligible APMs program as authorized under MACRA. The letter addressed numerous aspects of the MACRA law, and how the implementation should be done to positively impact the subspecialty of gynecologic oncology and those Medicare patients for whom SGO members provide care (46). SGO specifically commented on the following challenges to the implementation of MIPS: reporting mechanisms for quality performance; data stratification; barriers to successful quality performance; data accuracy; resource use performance; Clinical Performance Improvement Activities (CPIA); development of performance standards; and defining and incorporating improvement and public reporting.

“The forthcoming regulations should establish an easy pathway for PFPM [Physician-Focused Payment Models] proposals to be adopted as qualified APMs. CMS should clearly outline the criteria that will be used to evaluate PFPM proposals. CMS and the [Physician Focused Payment Model Technical Advisory Committee] PTAC should work collaboratively with medical societies and other organizations developing proposals, provide feedback on drafts, and provide data up-front to help in modeling impacts. These regulations should also make it clear that PFPMs recommended by the PTAC will be accepted by CMS. SGO is working very hard on its endometrial cancer APM with the intent of having it accepted as a PFPM” (46).

The SGO has endorsed disease site-specific quality indicators for ovarian, endometrial, and cervical cancers (47). In December 2015, CMS selected for consideration nine of the 15 process measures specific for gynecologic oncology (48). These quality measures were submitted for possible inclusion in the PQRS for 2017, which will be the first reporting year for MIPS. Should SGO’s measures be accepted, they will be published in the CY 2017 Proposed Medicare Physician Fee Schedule Rule and will again be open for comment.

## Discussion

The payment reforms underway are intended to drive the improvement of patient-centered, high-quality, and efficient care that is consistent with the Institute of Medicine’s six aims (49). The future of payment reform centers on legislation that incentives participation in an APM, while creating an environment where FFS is less tenable. However, a successful transition from the current state to an APM assumes that the new paradigm will be clearly defined, equitable, and flexible enough to accommodate the necessary variation and heterogeneous environments in which oncology is practiced today. Medical societies and engaged physicians will certainly be critical in creating meaningful, actionable, and measurable quality metrics that will be important components of MIPS. The SGO has taken critical steps to develop and implement a Clinical Outcomes Registry (50). This sub-specialty-specific registry was designed to be a tool to measure quality, compare outcomes, and could function as a platform for quality improvement and outcomes research.

Depending on a particular current state, moving into an APM will require a variable level of practice and culture transformation. For example, although dynamic gynecologic oncology-specific pathways have been developed, assessment of compliance is currently a resource intense process. Technology solutions, such as clinical decision support, may improve pathway monitoring but must also allow for necessary variation unique to cancer patients. Transforming practices into APMs beyond pathways will require local solutions that demand insight of the practice environment and strategic decisions that must account for many factors. Such critical factors include degree of physician integration; scope of oncology services provided; scale of health care system; financial health of the involved practice(s), hospital(s), and payer(s); competitive landscape of local market; and risk tolerance of the enterprise(s). Each practice environment will face unique challenges to adaptation. For example, smaller single-specialty practices may need to vie for scale or develop strategic partnerships to optimize care coordination. Larger academic practices with research and education components to their mission may be stressed as margins tighten. It will be incumbent on gynecologic oncologists and academic institutions to structure APMs such that gynecologic cancer research and education can be sustainable and continue to advance the field into the future.

Gynecologic oncologists function in a very wide array of practice settings. Therefore, the design of APMs will require flexibility. APMs impose both an administrative burden and financial risk that is likely to accelerate the existing trend toward practice consolidation. Independent, physician-owned practices may lack the resources, scope, and scale required to achieve and sustain compliance with the added administrative burdens involved in APM participation. Smaller practices are also less likely to be able to absorb the potential losses in a model that involves downside financial risk. Even gynecologic oncologists who are already employed or part of a large physician group will be affected. The hospitals or practices that employ gynecologic oncologists may further consolidate to achieve scale, which may further affect the employed physicians. No doubt, multi-specialty oncology practices and hospitals will be developing APMs for a wide range of disease sites, which may make cross-comparisons of gynecologic cancer APMs difficult between different geographic regions. The transformation of current models into a value-driven framework may require solutions devised at the local level. That is, how the new APM is developed and deployed may vary widely depending on the specific practice environment of a gynecologic oncologist. As these changes unfold, it will be critical that gynecologic cancer care remains patient-centered and of the highest quality.

The upside to the provision of high-quality, accountable, patient-centered care is clear. However, there are increasing resource-intensive administrative components to practice which must be considered as innovative payment models are designed. Some of these requirements divert time and resources away from direct patient care. According to a recent commentary (51), “the quality-measurement enterprise in U.S. health care is troubled.” Some physicians, hospitals, and health plans view measurement as burdensome, expensive, inaccurate, and indifferent to the complexity of care delivery. Although P4P programs are among the oldest APMs, the success of these models is impeded by serious gaps in the current quality measurement system. According to a 2014 RAND report (52) that looked at 49 studies examining the effect of P4P on process and intermediate outcome measures, the overall results of the studies were mixed, and any identified effects were relatively small. A basic flaw in the design of existing P4P models is the reality that meaningful oncology-specific patient-centered outcome measures remain elusive. Although the pursuit of better value in cancer care is a necessary goal, simply establishing timelines is inadequate. Even with the repeal of the SGR, there are major challenges to achieving value-driven cancer care, including the lack of an agreed-upon, patient-centered definition of value; a shortage of meaningful and actionable performance metrics; and a deficiency of accounting systems capable of reflecting the true total cost of delivering cancer care.

## Conclusion

Substantive payment reform in oncology is timely because there is great opportunity to align payments with the triple aim of better health and better care at a lower cost. The models described represent potential ways to address deficiencies in the current system, such as high and variable spending, fragmented and uncoordinated care, and insufficient reimbursement for services that often make a difference for patients and their families. APMs vary in the size, scope, and degree to which they shift away from FFS, but they increase provider accountability and support for innovative care delivery components.

While unprecedented payment reform activity is taking place in oncology, results are limited, and more evidence is needed to fully understand the implications of MACRA, MIPS, and APMs. To date, there are anecdotal examples of APM pilots around the country, but widespread adoption of new APMs by multiple payers is essential to build the evidence in support of a model. Although surgical oncology and radiation oncology pilots exist, most APMs to date have focused predominantly on medical oncology. Cancer care is far more interdisciplinary, and the most forward-thinking APMs must aim to incorporate the totality of care for the cancer patient. Given the range of services provided by gynecologic oncologists, payment reform has the potential to disrupt this sub-specialty disproportionately. Analysis and reporting of the initial experiences will be important to learn and make iterative improvements. While striving for breakthrough innovation, the path forward may require some degree of experimentation and tolerance for failure by all parties involved. Preliminary experience indicates that savings can be achieved by payment reforms that support increased care coordination and the greater use of physician-led care teams. For example, such initiatives can reduce hospital readmissions, complications, and unnecessary imaging. Therefore, a critical priority is to develop further evidence of how new payment systems in oncology can better align physician reimbursement with care transformations to improve care coordination, quality of care, population health, and the patient experience. Engagement in payment reform is a unique opportunity to positively impact the future state of gynecologic oncology. In addition to fiscally responsible high-quality patient care, efforts in reform must protect research and education as margins tighten. Although much remains unknown, focused attention of gynecologic oncologists on payment reform is imperative to assure that the practice remains patient centered, embodies the highest quality, yet transforms into a sustainable and agile sub-specialty to pro-actively and effectively manage the immense and relentless financial pressures and regulatory expectations to come.

## Author Contributions

SA was the primary author of the manuscript, including planning, research, writing, and review. KP provided expert review and feedback.

## Conflict of Interest Statement

The authors declare that the research was conducted in the absence of any commercial or financial relationships that could be construed as a potential conflict of interest.
